# Low bone mineral density is found in low weight female youth with avoidant/restrictive food intake disorder and associated with higher PYY levels

**DOI:** 10.1186/s40337-023-00822-y

**Published:** 2023-07-01

**Authors:** Aluma Chovel Sella, Kendra R. Becker, Meghan Slattery, Kristine Hauser, Elisa Asanza, Casey Stern, Megan Kuhnle, Nadia Micali, Kamryn T. Eddy, Madhusmita Misra, Jennifer J. Thomas, Elizabeth A. Lawson

**Affiliations:** 1 Neuroendocrine Unit, Massachusetts General Hospital, 55 Fruit St., Boston, MA 02114 USA; 2 Division of Pediatric Endocrinology, Mass General Hospital for Children, Boston, MA USA; 3 Eating Disorders Clinical and Research Program, Massachusetts General Hospital, Boston, MA USA; 4 Department of Psychiatry, Harvard Medical School, Boston, MA USA; 5 Great Ormond Street Institute of Child Health, University College London, London, UK; 6 Department of Pediatrics Gynecology and Obstetrics, Faculty of Medicine, University of Geneva, Geneva, Switzerland; 7 Department of Pediatrics, Harvard Medical School, Boston, MA USA; 8 Department of Medicine, Harvard Medical School, Boston, MA USA; 9 Mental Health Services in the Capital Region of Denmark, Eating Disorders Research Unit, Psychiatric Centre Ballerup, Ballerup, Denmark

**Keywords:** Adolescence, ARFID, Avoidant/restrictive food intake disorder, Bone health, Bone mineral Density, BMD, DXA, Feeding and eating disorder, Low weight, PYY

## Abstract

**Background:**

Avoidant/restrictive food intake disorder (ARFID) is a restrictive eating disorder commonly associated with medical complications of undernutrition and low weight. In adolescence, a critical time for bone accrual, the impact of ARFID on bone health is uncertain. We aimed to study bone health in low-weight females with ARFID, as well as the association between peptide YY (PYY), an anorexigenic hormone with a role in regulation of bone metabolism, and bone mineral density (BMD) in these individuals. We hypothesized that BMD would be lower in low-weight females with ARFID than healthy controls (HC), and that PYY levels would be negatively associated with BMD.

**Methods:**

We performed a cross-sectional study in 14 adolescent low-weight females with ARFID and 20 HC 10–23 years old. We assessed BMD (total body, total body less head and lumbar spine) using dual x-ray absorptiometry (DXA) and assessed fasting total PYY concentration in blood.

**Results:**

Total body BMD Z-scores were significantly lower in ARFID than in HC (− 1.41 ± 0.28 vs. − 0.50 ± 0.25, *p* = 0.021). Mean PYY levels trended higher in ARFID vs. HC (98.18 ± 13.55 pg/ml vs. 71.40 ± 5.61 pg/ml, *p* = 0.055). In multivariate analysis within the ARFID group, PYY was negatively associated with lumbar BMD adjusted for age (*β* = -0.481, *p* = 0.032).

**Conclusion:**

Our findings suggest that female adolescents with low-weight ARFID may have lower BMD than healthy controls and that higher PYY levels may be associated with lower BMD at some, but not all, sites in ARFID. Further research with larger samples will be important to investigate whether high PYY drives bone loss in ARFID.

## Abstract (plain language summary)

Avoidant/restrictive food Intake disorder (ARFID) is a condition characterized by lack of interest in eating/food, sensory sensitivity and/or fear of aversive consequences of eating. It is associated with low weight and undernutrition, which can lead to medical complications. Specifically, low weight in patients with ARFID raises concerns of impaired bone health. In this study, we compared bone mineral density (BMD), a measure of bone health, in 14 low-weight females with ARFID and 20 healthy females 10–23 years old. We also examined the association between BMD and peptide YY (PYY), a hormone that induces satiety and inhibits bone formation. A strong negative association between bone health and PYY was previously reported in females with anorexia nervosa. Thus, we hypothesized a similar association in low weight females with ARFID. We found that BMD may be lower in low-weight females with ARFID than in healthy females and that higher PYY levels are associated with lower BMD at some but not all sites. We concluded that bone health may be a concern in low-weight females with ARFID. This finding is important as low BMD raises concerns for increased fracture risk, which in turn could have a detrimental effect on quality of life.

## Introduction

Avoidant/restrictive food intake disorder (ARFID) is characterized by a lack of interest in eating or food, sensory sensitivity, and/or a fear of aversive consequences of eating; as opposed to the body image disturbance and fear of weight gain that characterize anorexia nervosa (AN) [1, 2]. Similar to AN, ARFID is associated with medical complications of malnutrition [3, 4]. AN carries a significant risk for multifactorial bone loss due to undernutrition and low weight [5, 6]. In one study, 41% of adolescents girls were found to have bone mineral density (BMD) Z-scores of less than -1 at any one site and an additional 11% had BMD Z-scores of less than -2 [5].

Adolescents with ARFID are commonly underweight and malnourished, similar to patients with AN [1, 7]. This raises concerns for negative effects on bone health, particularly because adolescence is a critical time for bone accrual with more than 80% of peak bone mass accrued by 18 years of age [8]. Little is known about BMD in ARFID, with only two studies to date exploring this topic. One showed lower BMD in low-weight adult males with ARFID versus healthy controls (HC)[9] and the second reported no significant lumbar BMD Z-scores differences between ARFID and AN. [10] However, no study thus far has compared young low-weight females with ARFID to HC. There is a need to address this knowledge gap.

Peptide YY (PYY) is a gut derived anorexigenic hormone linked to bone loss in AN [11, 12]. PYY is secreted primarily by neuroendocrine L cells of the distal gut in response to food ingestion and promotes satiety by binding to the hypothalamic Y2 receptors of neuropeptide Y [13]. In rodents, selective deletion of the PYY receptor, Y1, in osteoblasts or of the Y2 receptor in the hypothalamus resulted in high bone mass indicating that PYY is a negative regulator of bone via activation of these receptors [13–15]. Specifically, deletion of the Y1 receptor in osteoblasts showed increased osteoblastic bone formation and mineralization rates via increased Runx2 and Osterix expression [16]. Further, previous studies have shown that females with AN have elevated PYY levels compared to normal weight individuals or those with obesity [11, 12] and demonstrated a strong inverse correlation between PYY and BMD [11, 12]. We recently reported that fasting and postprandial levels of PYY in low-weight females with ARFID did not differ from HC or females with AN of similar low weight. Such PYY levels would be considered to be maladaptively high for a satiety inducing hormone in low-weight individuals, as decreased anorexigenic signaling would typically be expected in an undernourished state [17].

Given these findings, we speculated that bone health of low-weight females with ARFID may be compromised by undernutrition. Additionally, inappropriately high levels of PYY in the setting of undernutrition may contribute to bone loss. In this first cross-sectional study of BMD in young low-weight females with ARFID compared to healthy normal-weight controls, we hypothesized that low-weight females with ARFID would have lower BMD, and that in the ARFID group, higher PYY levels would be associated with lower BMD.

## Methods

### Design

Participants were drawn from National Institutes of Health (NIH) funded studies evaluating the neurobiology of eating disorders (R01MH103402, Apr 2014–Mar 2020; R01MH108595, Mar 2016–Feb 2021; participants with ARFID and HC), bone health in AN (R01DK062249, July 2003–Feb 2011; HC), and endocrine function in young athletes (R01HD60827, Sep 2009–Jun 2016; HC).

For participants < 18 years old, written consent was signed by a parent/guardian and assent by the participant. Participants ≥ 18 years signed written consent. All study procedures were approved by the Mass General Brigham Institutional Review Board. Participants were seen at the Massachusetts General Hospital (MGH) Translational and Clinical Research Center and at the MGH/Harvard-Massachusetts Institute of Technology Division of Health Science and Technology Martinos Center for Biomedical Imaging.

### Subjects

We studied 14 low-weight females with ARFID and 20 HC aged 10–23 years. The diagnosis of ARFID was confirmed by one of the following:

1. The Kiddie Schedule for Affective Disorder and Schizophrenia—Present and Lifetime (KSADS-PL) [18] (for studies R01MH108595 and R01MH103402). This is a semi-structured interview that generates DSM-5 Axis I diagnoses, including feeding and eating disorders, for youths. In both studies, we used the KSADS to rule out eating disorders other than ARFID, and to assess restrictive eating behaviors consistent with ARFID.
2. The Eating Disorder Assessment for DSM-5 (EDA-5) (in study R01MH108595). This is a semi-structured interview specifically developed to derive DSM-5 feeding and eating disorder diagnoses, including ARFID [19].
3. The Pica, ARFID, and Rumination Disorder Interview (PARDI) [20] (in study R01MH108595). This is a semi-structured interview that can be used to confer ARFID diagnoses and assess severity and related impairment.
4. The Eating Disorder Examination (EDE) Version 17.0 [21] (in study R01MH103402). This is a semi-structured clinical interview used to confer DSM-5 feeding and eating disorder diagnoses. In R01MH103402 we used the EDE to rule out eating disorders other than ARFID, and to assess restrictive eating behaviors consistent with ARFID.

By study definition, all subjects with low-weight ARFID had ≤ 90% of expected body weight (EBW) determined by body mass index (BMI) divided by 50^th^ percentile of BMI for age; or ≤ 90% of EBW for height [17] and did not meet criteria for any other eating disorder. HC were required to have a BMI between the 10th to 90th percentiles, no pubertal delay (pubertal delay defined as menarche at > 16 years or thelarche at > 13 years) and regular menstrual periods (i.e. 9 or more menses over 12 months), if ≥ 2 years post-menarche.

Exclusion criteria for all participants included hematocrit < 30%, active pregnancy or current breastfeeding, use of systemic hormones or oral contraceptives within 8 weeks of enrollment, history of psychosis, active suicidal ideation, active substance or alcohol use disorder, intellectual disability (IQ < 70), any significant illness that the investigator determined could interfere with the study and impact data collection or participant safety, contraindications to MRI or inability to tolerate being in the MRI machine for an hour (due to other analyses done in these parent studies).

Additional exclusion criteria for HC (depending on the study they were drawn from) included potassium < 3 mmol/l, glucose < 50 mg/dl (R01 DK062249); elevated FSH (R01 DK062249 and R01 HD60827); use of medications or concurrent diseases known to affect bone metabolism (R01 DK062249 and R01 HD60827); bone fracture within 6 months of the study (R01 DK062249); history of migraines, thromboembolism, smoking and a first-degree relative with breast cancer (R01 HD60827); migraines with aura (R01MH103402); vegetarianism, familial history of anorexia nervosa or other low weight eating disorders in first degree relatives (R01MH103402); gastrointestinal surgeries, a lifetime history of psychiatric disorder by KSADS-PL, any feeding or eating disorder as assessed via EDA-5 or by history (R01MH108595, R01MH103402).

### Study procedures

Following informed consent, participants were screened to determine eligibility. The screening and baseline visits included a detailed medical history, physical examination including measurements of height, weight, and Tanner staging, followed by a blood sample to rule out anemia, urine βHCG to rule out pregnancy and an assessment of psychopathology by the KSAD-PL, PARDI, or EDE (R01MH103402, R01MH108595).

For eligible participants, we performed an assessment of bone health by dual-energy x-ray absorptiometry (DXA) at the baseline visit or at a main study visit, or at a separate visit for DXA. All participants obtained a blood sample for PYY following an overnight fast.

### Biochemical and bone mineral density analyses

To assess fasting plasma PYY levels, we collected whole blood in EDTA-plasma tubes and placed on ice. Plasma was separated and stored at -80 ºC. PYY levels were analyzed at the Brigham Assay Core Laboratory using an enzyme linked immunosorbent assay [Millipore Corporation; intra-assay coefficient of variation (CV) 17–18%, inter-assay CV 12–18%, lowest reportable value 10 pg/ml with dynamic range 10–2000 pg/ml)].

We assessed BMD and BMD Z-scores of the lumbar spine, total body less head, and total body, the three recommended sites for evaluating bone health in children and adolescents, by DXA using Hologic QDR-Horizon A, software version 13.6.0.4 and 13.6.0.5; Hologic Inc., Waltham, Massachusetts (MA), and by the Hologic QDR-Discovery A, software versions 13.3 and 13.5.3.2; Hologic Inc., Waltham, MA. From DXA reports, we extracted BMD Z-scores for the lumbar spine and total body based on means and standard deviations for age, sex and race available in the Hologic and Discovery A database. This information was not available for the total body less head for all subjects. Thus, BMD Z-scores for total body less head were obtained using the Zemel calculator based on data from the longitudinal Bone Mineral Density in Childhood Study [22]. Because this calculator allows BMD Z-score assessment for individuals ≤ 20 years old only, total body less head BMD Z-scores are not available for 3 ARFID and 4 HC participants, who were ≥ 21 years old.

### Statistical analysis

We compared demographic and clinical characteristics across the ARFID and HC groups using the Student *t*-test (as all variables were normally distributed) for continuous variables and Chi Square for categorial variables. We present all continuous variables as mean ± standard error of the mean (SEM) and all categorical data as count (%). We used linear regression to determine associations between BMD and PYY levels in low weight females with ARFID, followed by multivariate analysis to control for age. We defined statistical significance as a two-tailed *p-*value < 0.05. We performed statistical analyses using JMP Pro 16.0.0 software.

## Results

Table 1 displays demographics and clinical characteristics. Low-weight females with ARFID and HC were 16.41 ± 0.96 and 16.29 ± 0.78 years old, respectively (*p* = 0.954), with no significant difference in Tanner staging for breasts or pubic hair (3.71 ± 0.30 vs 4.05 ± 0.28, *p* = 0.436 and 3.64 ± 0.32 vs 4.05 ± 0.28, *p* = 0.358, respectively). Per protocol definitions, subjects with ARFID had lower BMI and BMI percentiles than HC. Total fat mass, as measured by DXA, was lower in ARFID compared to HC. Mean PYY levels trended higher in ARFID versus HC (Table 1). Medical comorbidities and/or medications potentially detrimental to bone density were not prevalent among subjects with ARFID. Four subjects (28%) had asthma without a history of chronic glucocorticoid use (inhaled or systemic). Regarding medications, two subjects (14%) were taking vitamin D supplements, two (14%) were on calcium supplements (with no known deficiencies) and four (28%) on multivitamins. Other medications included anti-depressants, stimulants for the treatment of attention deficit/hyperactivity disorder, a proton pump inhibitor (Omeprazole), and cyproheptadine to increase appetite.

**Table 1:** Demographic and clinical characteristics in ARFID vs. healthy control groups

Clinical characteristics	ARFID (*n* = 14)	HC (*n* = 20)	*p* value
Age (years)	16.41 ± 0.96	16.29 ± 0.78	0.954
Race, *n* (%)			0.525
*Asian*	1 (7.14)	1 (5.00)	
*Black or African American*	0	0	
*White*	11 (78.58)	15 (75.00)	
*More than one race*	2 (14.28)	4 (20.00)	
Premenarchal	6 (42.86)	4 (20.00)	0.252
Total exposure to estrogen in past 9 months (months)**	4.79 ± 1.20	6.75 ± 3.64	0.183
Tanner stage (breasts)	3.71 ± 0.30	4.05 ± 0.28	0.436
Tanner stage (pubic hair)	3.64 ± 0.32	4.05 ± 0.28	0.358
Weight (kg)	40.54 ± 1.63	56.60 ± 2.44	< 0.001*
Height (cm)	157.36 ± 2.19	161.72 ± 2.04	0.163
BMI (kg/m^2^)	16.27 ± 0.27	21.45 ± 0.61	< 0.001*
BMI z-score	− 1.71 ± 0.25 (*n* = 11)	0.29 ± 0.17 (*n* = 16)	< 0.001*
Total fat mass (kg)	11.14 ± 0.59	16.01 ± 1.42	0.010*
Total lean mass (kg)	28.83 ± 1.18	33.90 ± 2.98	0.211
% Body fat	26.40 ± 0.69	31.67 ± 1.10	< 0.001*
PYY (pg/ml)	98.18 ± 13.55 (*n* = 11)	71.40 ± 5.61 (*n* = 15)	0.055
Total body BMD (g/cm^2^)	0.93 ± 0.03	1.00 ± 0.03	0.105
Total body less head BMD (g/cm^2^)	0.81 ± 0.09	0.88 ± 0.03	0.091
Lumbar spine BMD (g/cm^2^)	0.84 ± 0.03 (*n* = 13)	0.87 ± 0.04 (*n* = 18)	0.570

Mean ± SEM for all values unless note otherwise

*Significant *p* value ≤ 0.05

**Total exposure to estrogen in the past 9 months refers to the number of months of exposure to estrogen at physiologic levels or months of use of oral contraceptives

*ARFID* Avoidant/restrictive food intake disorder, *BMI* Body mass index, *HC* Healthy controls, *PYY* peptide YY

**Fig. 1:**
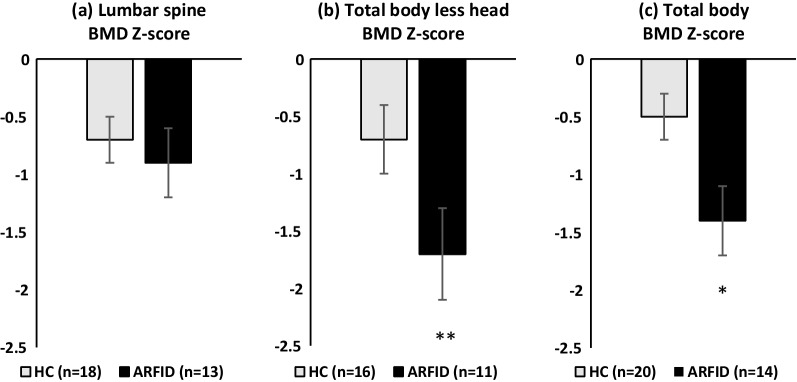
Bone mineral density (BMD) Z-scores for the **a** lumbar spine, **b** total body less head and **c** total body in low weight females with ARFID (black bars) and HC subjects (gray bars). *p = 0.021; **p = 0.055

Bone variables are presented in Table 1 and Fig. 1. Total body BMD Z-scores were significantly lower in ARFID vs. HC (− 1.41 ± 0.28 vs − 0.50 ± 0.25 respectively, *p* = 0.021) and total body less head BMD Z-scores trended lower in ARFID versus HC (− 1.67 ± 0.40 vs − 0.74 ± 0.27 respectively, *p* = 0.055). Lumbar BMD Z-scores were numerically lower in ARFID vs. HC; however, this did not reach statistical significance (− 0.95 ± 0.35 vs. − 0.67 ± 0.23 respectively, *p* = 0.489).

**Fig. 2:**
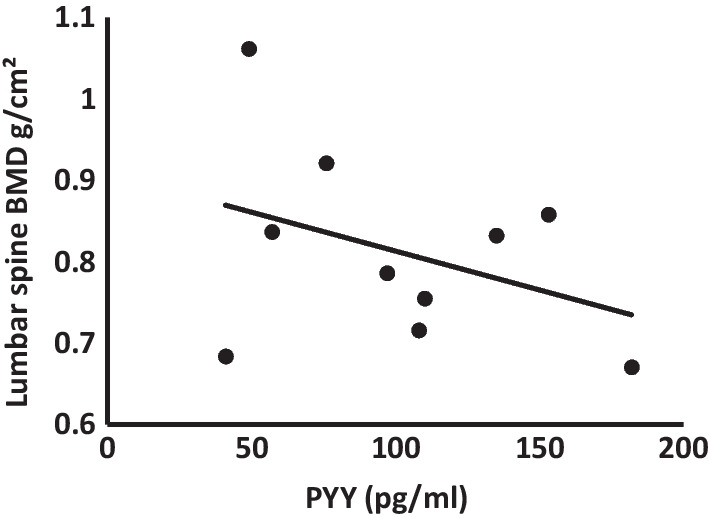
Correlation between PYY levels and age-adjusted lumbar spine BMD in subjects with ARFID. Among low weight females with ARFID who also had PYY data (*n* = 10), PYY levels were negatively correlated with age-adjusted lumbar BMD (*β* = -0.481, *p* = 0.032) on multivariate analysis. *BMD* Bone mineral density; *PYY* peptide YY

Among low weight females with ARFID who also had PYY data (*n* = 10), PYY levels were negatively correlated with age-adjusted lumbar BMD (*β* = -0.481, *p* = 0.032) on multivariate analysis (Fig. 2). We did not find correlations between PYY levels and total body BMD or total body less head BMD (*p* = 0.55 and *p* = 0.72 respectively).

## Discussion

This study was the first to compare bone health in low-weight females with ARFID versus HC. We found lower total body BMD Z-scores in those with ARFID, suggesting that bone health may be compromised in this disorder. Low BMD carries a long-term risk of fractures as well as a potentially detrimental effect on quality of life [23]. Ultimately, this line of research may lead to further investigation of bone health in ARFID, potentially leading to establishment of guidelines for screening for bone health in young patients with ARFID. Further, consistent with our hypothesis, we found that higher levels of PYY (a hormone that inhibits osteoblast activity) were associated with lower BMD (when adjusted for age) among subjects with ARFID, though this association was noted for the lumbar spine and not for the total body.

Low BMD is an established concern in young females with AN, with up to 52% of adolescents having a BMD Z -score of < − 1 at one or more sites. Further, an increased risk of fractures is a significant concern among these individuals [5]. In contrast, little is known regarding bone health in individuals with ARFID. An earlier study of 134 young males and females (comparing 118 with AN versus 16 with ARFID) did not show significant differences in BMD between the groups, suggesting that individuals with ARFID may be at similar risk for low BMD compared to individuals with AN [10]. Our study demonstrating lower total BMD Z-scores in low-weight subjects with ARFID compared to HC provides further evidence for the finding that low BMD is a concern in ARFID.

Similar to a previous report from our group [17], in the current study, PYY levels in subjects with ARFID did not significantly differ from HC despite lower weight in ARFID, where one would typically expect suppressed levels of hormones that signal satiety. In fact, PYY levels trended higher in low-weight girls with ARFID than in HC. Furthermore, higher PYY levels were associated with lower age-adjusted lumbar BMD in ARFID, consistent with prior findings in AN [11], and with PYY’s known inhibitory effect on bone formation. Interestingly, this association was observed for the lumbar spine, but not for total body BMD. Given that the lumbar spine is mostly trabecular bone, while the total body includes both cortical and trabecular bone, but mostly reflects cortical sites, our findings suggest that higher PYY levels in ARFID might have a deleterious impact preferentially at trabecular sites. This finding is supported by previous studies showing a twofold increase in trabecular bone volume but no significant increase in cortical bone in Y2 receptor knockout mice [24], as well as strong inverse correlations between PYY and BMD, particularly at the spine in women with AN [11]. Further investigation and larger studies are needed to confirm these findings.

Low BMD in AN is considered multifactorial and related to lower BMI, lower lean mass [5, 25], hypogonadism [5, 26], Growth Hormone resistance and low IGF-1 [27], high cortisol levels, high PYY levels [28], and low levels of leptin, oxytocin [29], insulin and amylin [30]. Of note, in this study despite no difference in estrogen exposure and no difference in lean mass across groups, total BMD Z-scores among subjects with ARFID were lower than in HC (*p* = 0.021). Total body less head BMD Z-scores also trended lower, but this was not statistically significant (*p* = 0.055). Our group has previously reported that low-weight adolescent females with ARFID have low leptin levels and lower IGF-1 Z-scores than HC [31], which could contribute to bone loss. The current study further indicates a potential role for PYY in low BMD in ARFID. Taken together, these data suggest that low BMD in ARFID, as in AN, may be multifactorial with potentially overlapping [17] and also distinctive contributing factors from AN. Further investigation of the pathophysiology of bone loss in ARFID will be important.

Our study has limitations. Given its cross-sectional design, causality cannot be assumed. Our sample size was small and may have underpowered our capability to identify statistically significant differences across groups, such as for lumbar spine BMD and certain clinical characteristics. In addition, not all subjects had PYY levels assessed and not all had lumbar BMD data. Lastly, serum calcium, phosphorus, alkaline phosphatase and 25-hydroxy vitamin D levels were not available for most of the subjects. Thus, larger studies are needed, potentially with broader inclusion criteria for ARFID (e.g. including males and individuals across the weight spectrum). In concordance with pediatric recommendations, we examined only whole body and lumbar spine BMD. A broader approach would be to include additional sites, such as the total hip and femoral neck, as was recently recommended by the International Society For Clinical Densitometry for older adolescents [32]. Future investigation of additional sites and using more advanced imaging for bone microstructure as well as markers of bone turnover will add to our understanding of bone health and fracture risk in individuals with ARFID.

In summary, bone health in low weight individuals with ARFID is of concern. Higher levels of PYY may promote multifactorial bone loss in low weight females with ARFID. Further investigation is warranted to improve our understanding of bone health in individuals with ARFID and to guide monitoring and treatment.

## Data Availability

The datasets used and/or analyzed during the current study are available from the corresponding author on reasonable request.

## Abbreviations

AN: Anorexia nervosa
ARFID: Avoidant/restrictive food intake disorder
BMI: Body mass index
BMD: Bone mineral density
CV: Coefficient of variation
DXA: Dual X-ray absorptiometry
EDA-5: Eating Disorder Assessment for DSM-5
EDE: Eating Disorder Examination
EBW: Expected body weight
HC: Healthy controls
KSADS-PL: Kiddie Schedule for Affective Disorders and Schizophrenia-present and lifetime
MGH: Massachusetts General Hospital
MA: Massachusetts
NIH: National Institutes of Health
PYY: Peptide YY
PARDI: Pica, ARFID, and Rumination Disorder Interview
SEM: Standard error of the mean
